# Association of genetic variants related to combined exposure to higher BMI and waist-to-hip ratio on lifelong cardiovascular risk in UK Biobank

**DOI:** 10.1017/S1368980022001276

**Published:** 2022-05-27

**Authors:** Eric Yuk Fai Wan, Wing Tung Fung, Esther Yee Tak Yu, Will Ho Gi Cheng, Kam Suen Chan, Yuan Wang, Esther Wai Yin Chan, Ian Chi Kei Wong, Cindy Lo Kuen Lam

**Affiliations:** 1 Department of Family Medicine and Primary Care, Li Ka Shing Faculty of Medicine, The University of Hong Kong, Hong Kong Special Administrative Region, China; 2 Centre for Safe Medication Practice and Research, Department of Pharmacology and Pharmacy, The University of Hong Kong, Hong Kong Special Administrative Region, China; 3 Laboratory of Data Discovery for Health (D^2^4H), Hong Kong Special Administrative Region, China; 4 Research Department of Practice and Policy, School of Pharmacy, University College London, London, UK; 5 Department of Family Medicine, The University of Hong Kong Shenzhen Hospital, Shenzhen, China

**Keywords:** Obesity, BMI, Waist-to–hip ratio, Genetic risk score, CVD, Cardiovascular risk

## Abstract

**Objective:**

This study examines the individual and combined association of BMI and waist-to-hip ratio (WHR) with CVD risk using genetic scores of the obesity measurements as proxies.

**Design:**

A 2 × 2 factorial analysis approach was applied, with participants divided into four groups of lifetime exposure to low BMI and WHR, high BMI, high WHR, and high BMI and WHR based on weighted genetic risk scores. The difference in CVD risk across groups was evaluated using multivariable logistic regression.

**Setting:**

Cohort study.

**Participants:**

A total of 408 003 participants were included from the prospective observational UK Biobank study.

**Results:**

A total of 58 429 CVD events were recorded. Compared to the low BMI and WHR genetic scores group, higher BMI or higher WHR genetic scores were associated with an increase in CVD risk (high WHR: OR, 1·07; 95 % CI (1·04, 1·10)); high BMI: OR, 1·12; 95 % CI (1·09, 1·16). A weak additive effect on CVD risk was found between BMI and WHR (high BMI and WHR: OR, 1·16; 95 % CI (1·12, 1·19)). Subgroup analysis showed similar patterns between different sex, age (<65, ≥65 years old), smoking status, Townsend deprivation index, fasting glucose level and medication uses, but lower systolic blood pressure was associated with higher CVD risk in obese participants.

**Conclusions:**

High BMI and WHR were associated with increased CVD risk, and their effects are weakly additive. Even though there were overlapping of effect, both BMI and WHR are important in assessing the CVD risk in the general population.

The worldwide prevalence of obesity is increasing rapidly. It has nearly tripled since 1975, and in 2016, there were more than 1·9 billion overweight or obese adults globally^(1,2)^. Given that obesity is one of the known risk factors associated with adverse health outcomes, such as CVD and mortality^(3)^, it is crucial to examine the individual and/or combined effects of using different measurements in the assessment of obesity-associated CVD risks.

BMI is the most common measure of the weight status of an individual. It is also the recommended measurement for determining CVD risks according to current guidelines on obesity management by the American College of Cardiology and the American Heart Association in 2013^(4)^. Hence, previous studies have predominantly investigated the causal relationship between obesity and CVD risks using BMI^(3,5–8)^. However, a previous study showed that patients who were defined as overweight by BMI might surprisingly have lower mortality rate than normally weighted patients^(9)^. Thus, waist-to-hip ratio (WHR), which focuses on abdominal adiposity and distribution of body fat, has then been suggested as an alternative measurement for assessing obesity-associated CVD risks^(10,11)^. Significant correlation between WHR and CVD risks has been supported in recent studies^(12,13)^. Nevertheless, there is still debate on the preferred measurement for determining the association between obesity and CVD risks^(14)^. More importantly, it is uncertain whether there are any additive effects or interactions on CVD risks if both BMI and WHR are used. A large study composed of 221 934 patients in seventeen countries claimed that the measurement of both BMI and WHR offered similar effects on CVD risks prediction when used in combination^(15)^, but studies are yet to identify any incremental effects of measuring WHR, on top of BMI, on CVD risk^(16,17)^.

Given the increased availability of genetic studies, such as genome-wide association studies, there is increasing evidence of the contribution of genetics to the variation of BMI and WHR. Studies on twins and families have shown that obesity is highly heritable, suggesting that 30–70 % of variation in body size is due to genetic factors^(18–20)^. Genetic risk score is one of the approaches to summarise the genetic effects of multiple risk genes on a given trait. Traditionally, observational studies measure BMI and WHR at a limited follow-up period and are prone to unmeasured confounders and measurement errors^(9–11,21)^. Using genetic risk scores as proxies, the long-term effects of increased BMI or WHR, which are infeasible to be measured in randomised controlled trials, can be estimated.

Therefore, the aim of this study is to determine the individual and/or combinational effects of BMI and WHR genetic scores associated with CVD risks. Understanding the association between BMI/WHR and CVD risk can inform the practices in obesity management.

## Method

### Study population

The UK Biobank is an ongoing prospective cohort study that collects phenotypic and genetic data from around 500 000 participants across the United Kingdom. Participants were recruited between 2006 and 2010 and consisted of mostly people of European ancestry. Details of the study protocol have been described elsewhere^(22,23)^. Participants with available genetic data and of self-reported and genetically validated White British ancestry were included in our analysis. Participants with missing genotyping rates ≥1 %, who had sex aneuploidy and genetic sex discordance, or who were related to at least one individual (kinship index > 0·088) were excluded.

### Instruments of randomisation

The BMI genetic score was constructed by a total of 670 genetic variants associated with BMI at genome-wide significance (*P* < 5·0 × 10^-9^) and in low linkage disequilibrium, as reported by a previous genome-wide association study in the Genetic Investigation of Anthropometric Traits (GIANT) Consortium^(24)^. The exposure allele was defined as the allele associated with higher BMI. A weighted genetic score was calculated for each participant in the UK Biobank from the total number of BMI-increasing alleles in the participant’s genotype, weighted by the genome-wide association study-reported association of each genetic variant with BMI/kg/m^2^. Similarly, weighted WHR genetic score was constructed using a total of 316 genetic variants associated with WHR at genome-wide significance and in low linkage disequilibrium. Participants with missing data for one or more variants in either genetic score were excluded.

### Outcomes

Primary outcome was the occurrence of CVD event, which was defined by International Classification of Diseases (ICD) 9 and 10, and UK Biobank self-reported outcomes (see online Supplemental Table 1). CVD mortality and sixteen cardiovascular conditions were also examined as secondary outcomes. The sixteen cardiovascular conditions include IHD and its subtypes (myocardial infarction, ST elevation myocardial infarction and non-ST elevation myocardial infarction, stable angina and unstable angina), stroke and its subtypes (ischemic stroke, intracerebral haemorrhage and subarachnoid haemorrhage), heart failure, transient ischemic attack, peripheral vascular disease, arrhythmia and conduction disorder (including atrial fibrillation), pulmonary embolism and deep vein thrombosis. Leukaemia was used as a negative control. All the outcomes were presented and processed as binary outcomes and retrieved from UK Biobank on 14 November 2020.

### Study design

**Fig. 1:**
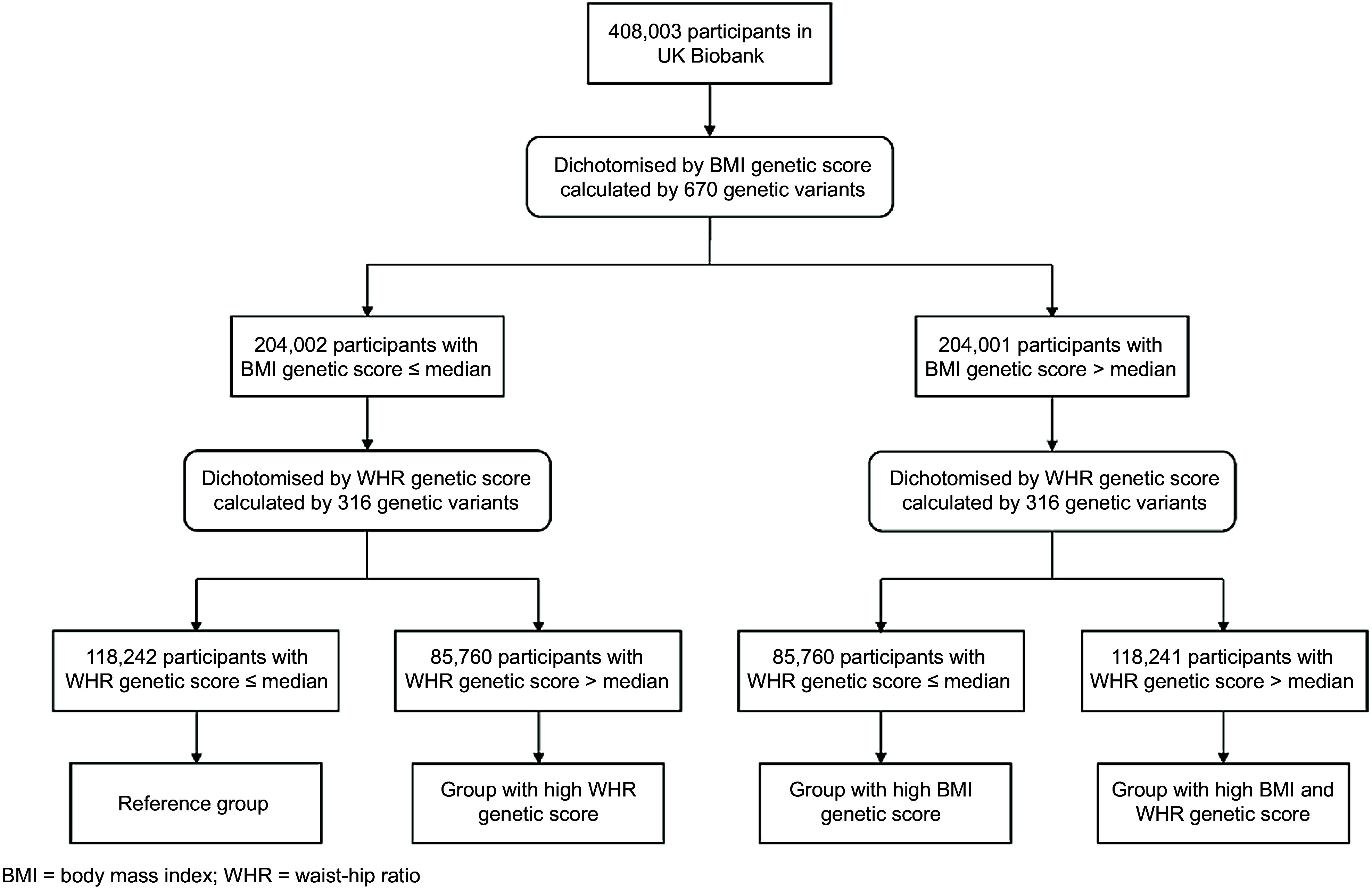
Study design schematic for using genetic scores as instruments of randomisation. WHR, waist-to-hip ratio

This study adopted a 2 × 2 factorial analysis, in which each dimension was the genetic score dichotomised by its median. The four resultant groups were groups with: (1) low BMI and WHR (reference group); (2) high BMI; (3) high WHR; and (4) high BMI and WHR genetic scores (Fig. 1).

### Statistical analysis

The relative CVD risks of groups with high BMI and/or high WHR genetic score to the reference group were estimated using multivariable logistic regression, adjusted with age, sex, current smoking status, Townsend deprivation index, LDL-cholesterol, fasting blood glucose, systolic blood pressure, diastolic blood pressure, and uses of antidiabetic drugs, antihypertensive drugs and lipid-lowering agents, which are established potential confounders of CVD^(25–27)^. Interaction between BMI and WHR genetic scores on CVD risk was evaluated using relative excess risk due to interaction (RERI), attributable proportion due to interaction (AP) and synergy index (S)^(28,29)^. Presence of interaction is indicated by RERI and AP larger than 0 and S larger than 1. Multivariable logistic regression was also performed to assess risks of CVD death and the sixteen CVD conditions among the four groups, as well as the association in various subgroups. The subgroups investigated included sex, age (≤65 and >65 years), current smoking status, Townsend deprivation index (most deprived: > 2·0, average: -1·9–2·0 and least deprived: ≤ -2·0), systolic blood pressure (<140 mmHg and ≥ 140 mmHg), fasting blood glucose (<7·0 mmol/l and ≥7·0 mmol/l), and uses of lipid-lowering agents, antihypertensive drugs or antidiabetic drugs. Interaction between genetic score groups and each subgroup was evaluated with likelihood ratio tests, indicated by *P*-value <0·05.

To assess the validity of the weighting approach used in genetic score calculation, sensitivity analyses were carried out using varying weightings, including unweighted genetic scores and genetic scores weighted by effect sizes from the UK Biobank data^(30)^. Additionally, an analysis was done using genetic score on WHR adjusted for BMI (WHRadjBMI), which represents another measure on body fat distribution^(24)^. To assess the validity of the dichotomisation cut-off, another sensitivity test was performed using means instead of medians as the cut-off. A 4 × 4 factorial analysis, in which participants were grouped based on genetic score quartiles, was also performed to evaluate the association of CVD risk and the magnitudes of the genetic scores at a finer scale.

## Results

A total of 408 003 participants were included, in which 45·9 % were male and the average age was 56·9 years (Table 1). There appeared to be a correlation between BMI and WHR genetic scores, as observed from the disproportion of participant number in the four groups. Participants were more likely to be in low BMI and WHR or high BMI and WHR groups than in the groups with either high BMI or high WHR. Participants with higher BMI or WHR genetic scores tend to have higher TAG, fasting blood glucose, and systolic and diastolic blood pressures and are more likely to be a smoker or a user of lipid-lowering agents, antihypertensive drugs or antidiabetic drugs.

**Table 1:** Baseline characteristics of participants by genetic risk score groups

	Overall		Low BMI and low WHR		Low BMI and high WHR		High BMI and low WHR		High BMI and high WHR	
	Mean	sd	Mean	sd	Mean	sd	Mean	sd	Mean	sd
No. of participants	408 003		118 242		85 760		85 760		118 241	
Age (year)	56·9	8·0	57·0	8·0	56·9	8·0	56·9	8·0	56·8	8·0
	*n*	%	*n*	%	*n*	%	*n*	%	*n*	%
Sex										
Male	187 441	45·9	53 907	45·6	39 200	45·7	39 670	46·3	54 664	46·2
Female	220 562	54·1	64 335	54·4	46 560	54·3	46 090	53·7	63 577	53·8
	Mean	sd	Mean	sd	Mean	sd	Mean	sd	Mean	sd
BMI (kg/m^2^)	27·4	4·8	26·3	4·2	26·6	4·3	28·2	5·0	28·6	5·0
WHR	0·87	0·09	0·86	0·09	0·87	0·09	0·87	0·09	0·89	0·09
	*n*	%	*n*	%	*n*	%	*n*	%	*n*	%
Smoker	41 186	10·1	10 764	9·1 %	8435	9·8	8903	10·4	13 084	11·1
	Mean	sd	Mean	sd	Mean	sd	Mean	sd	Mean	sd
Townsend deprivation index	−1·6	2·9	−1·66	2·87	−1·57	2·93	−1·54	2·94	−1·45	2·99
Lipids, mg/dl										
Total cholesterol	5·7	1·1	5·74	1·13	5·75	1·15	5·69	1·14	5·68	1·16
HDL-cholesterol	1·5	0·4	1·49	0·39	1·46	0·38	1·45	0·38	1·41	0·37
LDL-cholesterol	3·6	0·9	3·58	0·86	3·59	0·87	3·55	0·87	3·56	0·88
TAG	1·8	1·0	1·66	0·97	1·78	1·05	1·73	1·00	1·86	1·07
Fasting blood glucose (mmol/l)	5·1	1·2	5·07	1·10	5·10	1·13	5·12	1·22	5·18	1·35
Blood pressure (mmHg)										
Systolic blood pressure	138·3	18·6	137·6	18·7	138·2	18·6	138·5	18·6	139·0	18·4
Diastolic blood pressure	82·3	10·1	81·7	10·1	82·1	10·1	82·5	10·1	82·9	10·1
eGFR (ml/min/1·73 m²)	113·4	21·6	113·5	21·3	113·6	21·7	113·0	21·7	113·5	21·8
	*n*	%	*n*	%	*n*	%	*n*	%	*n*	%
Use of lipid-lowering agents	73 500	18·0	18 608	15·7	15 290	17·8	15 490	18·1	24 112	20·4
Use of antihypertensive drugs	41 277	10·1	10 783	9·1	8320	9·7	8987	10·5	13 187	11·2
Use of antidiabetic drugs	14 007	3·4	2856	2·4	2581	3·0	3015	3·5	5555	4·7

WHR, waist-to-hip ratio; eGFR, estimated glomerular filtration rate.

All values are presented in either mean (sd) or number (percentage).

**Fig. 2:**
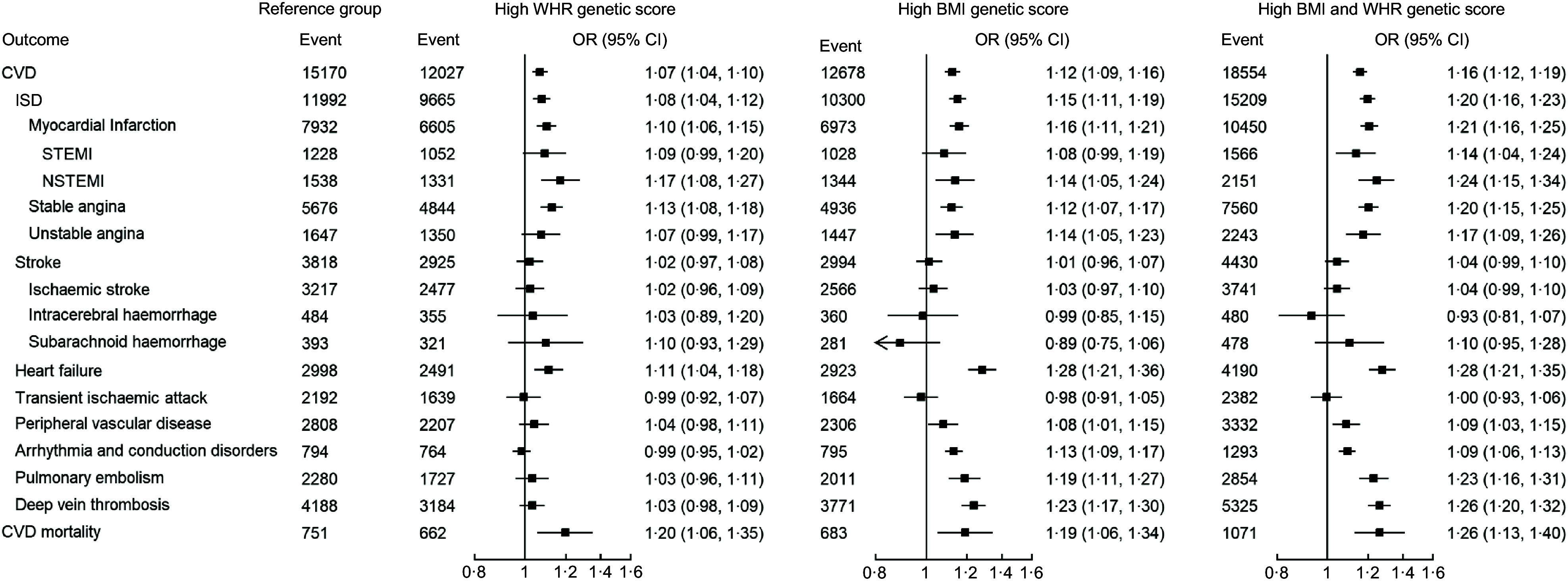
Association of exposure to higher BMI and WHR genetic score with cardiovascular outcomes. All logistic regression analyses were adjusted with sex, age, smoking status, Townsend deprivation index, LDL-cholesterol, fasting blood glucose, systolic blood pressure, diastolic blood pressure, and uses of antidiabetic drugs, antihypertensive drugs and lipid-lowering agents using the group of low BMI and low WHR as the reference. WHR, waist-to-hip ratio; NSTEMI, non-ST elevation myocardial infarction; STEMI, ST elevation myocardial infarction

The association between the genetic score groups and various cardiovascular outcomes is presented in Fig. 2. A total of 58 429 CVD events were recorded. Participants with high BMI or WHR genetic score were found to be more susceptible to CVD (high WHR: OR 1·07; 95 % CI (1·04, 1·10)); high BMI (OR 1·12; 95 % CI (1·09, 1·16)). A weak additive effect on CVD risk was observed, with the OR in the high BMI and WHR group exceeded the risk of the high genetic score group of each individual factor, but less than sum of the two (OR 1·16; 95 % CI (1·12, 1·19)). Similar trends were also observed in the various cardiovascular conditions investigated. Among the sixteen cardiovascular conditions, transient ischemic attack and stroke (overall and all subtypes) were the few conditions where no significant increase in risk in the high BMI and WHR group was observed. High BMI and WHR genetic scores were also found to be associated with increase in CVD mortality. In the assessment of interactions between BMI and WHR, the RERI, AP and S were -0·035 (95 % CI (-0·081, 0·011)), -0·030 (95 % CI (-0·069, 0·008)) and 0·82 (95 % CI (0·61, 1·03)), respectively, indicating the presence of a weak additive effect but the absence of interaction of BMI and WHR on CVD risk.

**Fig. 3:**
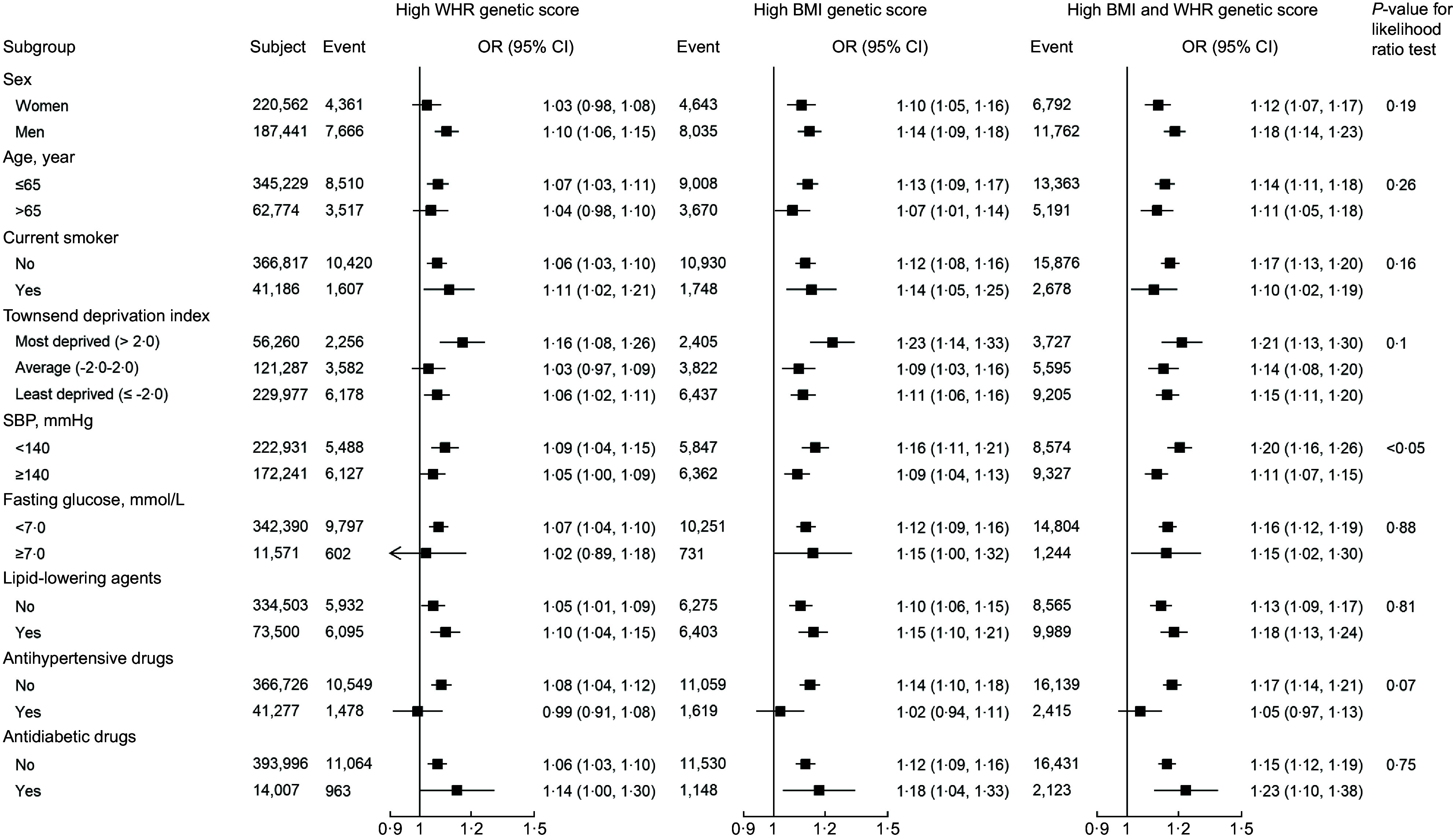
Association of exposure to higher BMI and WHR genetic score with cardiovascular events within subgroups. Logistic regressions were adjusted with sex, age, smoking status, Townsend deprivation index, LDL-cholesterol, fasting blood glucose, systolic blood pressure, diastolic blood pressure, and uses of antidiabetic drugs, antihypertensive drugs and lipid-lowering agents using the group of low BMI and low WHR genetic score as reference. WHR, waist-to-hip ratio

In subgroup analysis, the insignificant *P*-values from likelihood ratio test indicated similar associations between the genetic scores and CVD risk regardless of participants’ sex, age group, current smoking status, Townsend deprivation index, fasting blood glucose, and uses of lipid-lowering agent, antihypertensive drugs or antidiabetic drug (Fig. 3). However, significant interaction was observed in subgroups of systolic blood pressure. High BMI/WHR individuals with systolic blood pressure less than 140 mmHg had higher CVD risk.

**Fig. 4:**
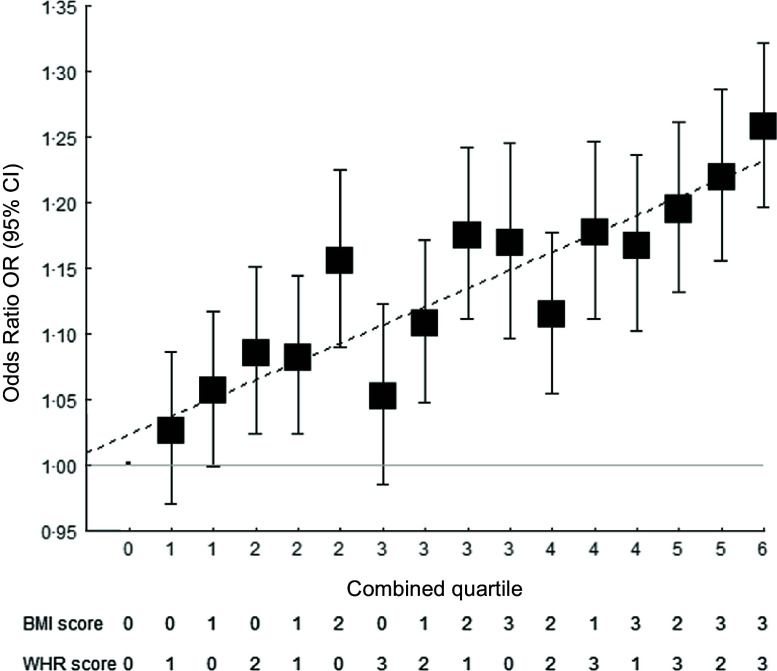
Association of high BMI and WHR genetic scores with CVD event stratified by quartiles. Logistic regressions were adjusted with sex, age, smoking status, Townsend deprivation index, LDL-cholesterol, fasting blood glucose, systolic blood pressure, diastolic blood pressure, and uses of antidiabetic drugs, antihypertensive drugs and lipid-lowering agents using the group at the lowest BMI and lowest WHR quartile as the reference group. WHR, waist-to-hip ratio

Sensitivity analyses using different genetic score calculations or cut-off presented similar associations of BMI and WHR on CVD risks (see online Supplemental Fig. 1), validating the genetic instruments used in the main analysis. As predicted, no association was found between the genetic scores and the negative control leukaemia. The 4 × 4 factorial analysis showed a gradual increase in CVD risk with increasing BMI and/or WHR genetic scores, with the highest CVD risk in individuals with both high BMI and WHR genetic scores (Fig. 4), suggesting an additive relation between the two.

## Discussion

Our analyses showed that genetic risk scores of BMI and WHR were associated strongly with various CVD events. When considering the genetic risk scores for both BMI and WHR, a weak additive effect with considerable overlapping on the CVD risk was observed. However, both BMI and WHR should be regarded as an independent risk factor for CVD.

Using either BMI or WHR, prior studies have demonstrated the individual effects of obesity and abdominal adiposity on the CVD risks, respectively^(5–8,12,13)^. Our results aligned with the established evidence on this causal relationship. Considering how both BMI and WHR could affect CVD risk, there is no consensus on the importance of each measure to CVD risks. A large-scale study has suggested that both adiposity measures share a similar strength of association with CVD^(15)^. Other studies reported uncertainty over the incremental effect of measuring fat distribution on the top of body mass on CVD risks^(16,17)^. Our study is the first to show a weak additive effect on the relationship of both BMI and WHR on CVD risks. While there is no recommendation on checking WHR in current guidelines for obesity management^(4)^, our finding suggests that BMI and WHR are equally important as biomarkers in early recognition, and thereafter, management of risk factors and prevention of CVD events.

It is well known that elevated BMI is associated with increased CVD risk. As body weight increases, the risk factors of CVD events, such as atherosclerosis, dyslipidaemia, hypertension and type 2 diabetes, are also found to increase^(12,31)^. However, there is a significant limitation on solely relying on BMI. As BMI measures the body mass of an individual as a whole, it omits other crucial risk factors of CVD, such as body composition and regional fat distribution^(32)^. For instance, conditions such as normal-weight central obesity would not have been picked up by BMI. In fact, normal-weight central obesity has been reported to associate with the highest risk of mortality among CVD patients^(33)^. Furthermore, it has been well established that central or visceral adiposity, independent of the body mass, is highly associated with CVD risk^(34–37)^. Together with our results, it implies that BMI and WHR are separate measures that focus on different aspects of obesity, and WHR has its own distinctive association with CVD risks irrespective of BMI. In short, their effects supplement each other additively, and the measurement of both BMI and WHR are therefore equally important.

Interestingly, our subgroup analysis revealed that the association between BMI/WHR and CVD risks is significantly stronger in the participants who had lower systolic blood pressure. The elevation of CVD risk by high BMI/WHR was more prominent in participants with low systolic blood pressure or who did not use antihypertensive drug. Although obesity is highly correlated with high blood pressure, they are independent risk factors of CVD^(38)^. Obese individuals with healthy metabolic status (including blood pressure, blood glucose and lipid profile) were still susceptible to higher risk in CVD than normal-weight individuals^(39,40)^. Some studies reported high blood pressure might be associated with more significant increase in CVD risk in normal-weight than obese individuals^(41,42)^, while some indicated a lack of difference^(43)^. The discrepancy observed could be because hypertension is linked to CVD through different mechanisms between normal-weight and overweight individuals^(44)^. Elevated blood pressure in normal-weight individuals might be more attributable to adverse lifestyle such as smoking and alcohol consumption^(45,46)^. Obesity in individuals with normal blood pressure could be a temporary state which is associated with younger age^(47)^. Effectiveness of antihypertensive drugs was also dependent on the patients’ weight^(48)^. Even though no significant difference in likelihood ratio test was observed in the antihypertensive drugs subgroup, it could be due to the relatively small samples of individuals taking antihypertensive drugs in our study. More in-depth study is needed to verify the role of hypertension in the association between high BMI/WHR and CVD.

While this study has established the independent association between CVD risks and BMI/WHR using genetic score proxies, one of the limitations is that it is uncertain how weight change by lifestyle or medical interference might affect the association. The results are, therefore, not representative for CVD risks due to BMI/WHR modifications by extrinsic factors, such as diet, exercises or medication. Moreover, as only Caucasians with British ancestry were included in this analysis, the result is not necessarily generalisable to other populations where the allele combinations might be vastly different from the UK dataset^(49,50)^. Finally, despite proving the importance of both obesity measures, our study is unable to provide a definite guideline on the optimal BMI/WHR threshold to be achieved for a reduction in CVD risk. Further studies are required for changes in clinical recommendations and practice.

## Conclusion

Our findings suggest that both BMI and WHR are associated with CVD risks independently, and there is a weak additive effect. The prominent association between BMI-/WHR-associated obesity and CVD risk among participants with lower blood pressure highlights the difference in susceptibility to chronic health problem across the population. As the role of BMI and WHR is not interchangeable in the causal relationship of obesity and CVD risks, both measurements should be recommended, in future guidelines for obesity management, especially for susceptible communities.

## Supplementary material

For supplementary material accompanying this paper visit https://doi.org/10.1017/S1368980022001276. click here to view supplementary material
