# Supplementary material for: Association of genetic variants related to combined exposure to higher BMI and waist-to-hip ratio on lifelong cardiovascular risk in UK Biobank
Source: Public Health Nutr. 2022 May 27;26(2):416–24. doi: 10.1017/S1368980022001276 (PMC13076085; doi:10.1017/S1368980022001276)
Supplement: Supplementary file 1 [file S1368980022001276sup.zip › S1368980022001276sup001.pdf]

Supplementary Table 1. Outcome definitions

Outcome	ICD-9 definitions	ICD-10 definitions	Self-reported codes
Cardiovascular disease	410.x, 411.x, 412.x, 413.x, 414.x, 429.79, 430.x, 431.x, 432.x, 433.x, 434.x, 435.x, 436.x, 437.x, 438.x	I20.x, I21.x, I22.x, I23.x, I24.1, I25.x, I46, I60.x, I61.x, I63.x, I64.x	1066, 1074, 1075, 1081, 1086, 1491, 1583
Ischemic heart disease	410.x - 414.x	I20.x - I25.x	1074, 1075
Myocardial Infarction	410.x, 411.x, 412.x, 429.79	I21.x, I22.x, I23.x, I24.1, I25.2	1075
STEMI	410.0 - 410.6, 410.8 - 410.9	I21.0 - I21.3, I22.0, I22.1, I22.8	N/A
NSTEMI	410.7	I21.4, I21.9, I22.9	N/A
Stable angina	413.1, 413.9	I20.1, I20.8, I20.9	N/A
Unstable angina	411.1, 411.81, 411.89	I20.0, I24.0, I24.8, I24.9	N/A
Stroke	430.x, 431.x, 432.x, 433.x, 434.x, 435.x, 436.x, 437.x, 438.x	I60.x, I61.x, I63.x, I64.x	1081, 1086, 1491, 1583
Ischaemic stroke	434.x,436.x	I63.x, I64.x	1583
Intracerebral haemorrhage	431.x	I61.x	1491
Subarachnoid haemorrhage	430.x	I60.x	1086
Heart failure	428.x	I50.x	1076
Transient ischemic attack	435.x	G45.0, G45.1, G45.2, G45.8, G45.9	1082
Peripheral vascular disease	250.6x, 440.2x, 443.1, 443.8, 443.9, 444.22, 444.81, 997.2, 997.6x	I73.x, I74.3, I74.4, I74.5	1067, 1088
Arrhythmia and conduction disorders (including atrial fibrillation)	426.x, 427.x	I44.x, I48.x	1077, 1471, 1483, 1484, 1485, 1486, 1487
Pulmonary embolism	415.1	I26	1093
Deep vein thrombosis	451.x - 453.x	I80 - I82	1068, 1094
Leukaemia	204.x, 205.x, 206.x, 207.x, 208.x, V10.6	C91.x, C92.x, C93.x, C94.0, C94.2, C94.3, C94.4, C94.8, C95.x, Z85.6	1048, 1055, 1056, 1074

ICD = International Classification of Diseases; NSTEMI = non-ST-elevation myocardial infarction; STEMI = ST-elevation myocardial infarction.

Supplementary Figure 1. Sensitivity test for association of exposure to high BMI and WHR genetic scores and cardiovascular diseases

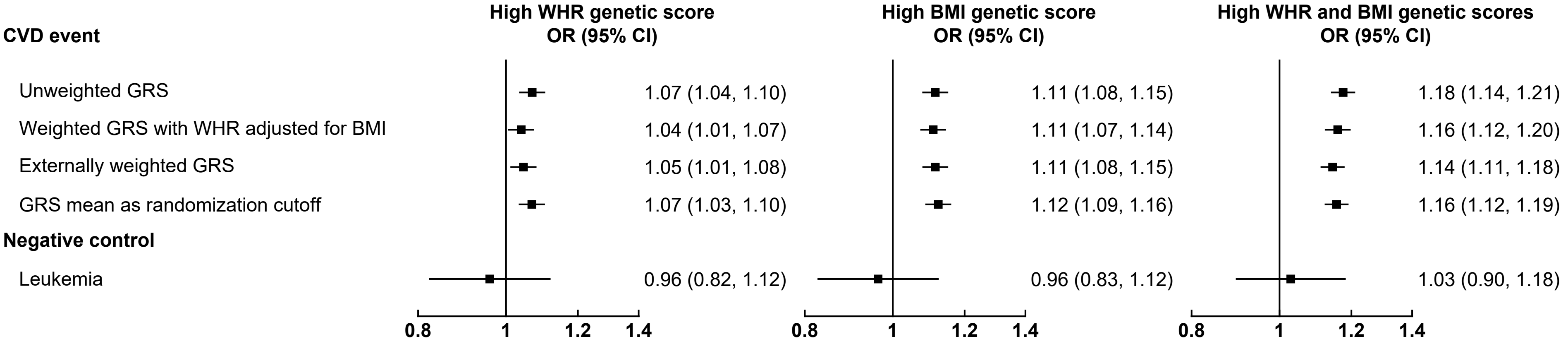

All logistic regression analyses were adjusted with sex, age, smoking status, Townsend deprivation index, low-density lipoprotein cholesterol, fasting blood glucose, systolic blood pressure, diastolic bloodpressure and uses of anti-diabetic drugs, anti-hypertensive drugs or lipid-lowering agents using the group of low BMI low WHR genetic scores as reference. CVD = cardiovascular diseases; BMI = body mass index; WHR = waist-hip ratio; GRS = genetic risk score; OR = odds ratio; CI = confidence interval.
